# Efficacy of Risankizumab across distinct PsA phenotypes identified with machine learning analytics using data from biologic DMARD-Naïve patients in two phase 3 clinical trials

**DOI:** 10.1186/s13075-025-03670-0

**Published:** 2025-11-29

**Authors:** Laure Gossec, Andra Balanescu, Maria Antonietta D’Agostino, Alexis Ogdie, Philipp Sewerin, Yu Deng, Linyu Shi, Yoshiyuki Sugimoto, Sheng Zhong, Yunzhao Xing, Ralph Lippe, Mitsumasa Kishimoto

**Affiliations:** 1 Sorbonne Université, INSERM, Institut Pierre Louis d’Epidémiologie et de Santé Publique, Team Pepites, Paris, France; 2 Rheumatology Department, AP-HP, Pitié-Salpêtrière hospital, Paris, France; 3 “Sf. Maria” Hospital, Department of Rheumatology and Internal Medicine, “Carol Davila” University of Medicine and Pharmacy, Bucharest, Romania; 4 Rheumatology Division, Department of Geriatrics, Rheumatology, and Orthopedic Sciences, Fondazione Policlinico Universitario Agostino Gemelli IRCCS, Rome, Italy; 5 Università Cattolica del Sacro Cuore, Largo Agostino Gemelli, Rome, Italy; 6 Department of Rheumatology, Perelman School of Medicine at the University of Pennsylvania, Philadelphia, PA USA; 7 Rheumazentrum Ruhrgebiet, Ruhr University Bochum, Bochum, Germany; 8 AbbVie Inc., North Chicago, IL USA; 9 AbbVie GK, Tokyo, Japan; 10 Department of Nephrology and Rheumatology, Kyorin University School of Medicine, Tokyo, Japan; 11 Service de Rhumatologie, Hôpital Pitié Salpétrière, 47-83, boulevard de l’Hôpital, Paris, 75013 France

**Keywords:** Biological therapy, Interleukin (IL)-23, KEEPsAKE, Machine learning, Phenotype, Psoriatic arthritis (PsA), Risankizumab

## Abstract

**Background:**

The development of personalized approaches in psoriatic arthritis (PsA) is challenging due to unclear patient phenotypes and trajectories. Machine learning (ML) could help to identify homogeneous patient groups. The objective of this analysis was to classify patients with PsA into distinct phenotypes using ML.

**Methods:**

A post hoc analysis of PsA patients treated with risankizumab for ≤ 4 years (196 weeks) from KEEPsAKE 1 and KEEPsAKE 2. The phenotypes were based on baseline demographics and clinical characteristics using unsupervised ML (a finite mixture model). Response to risankizumab at 4 years was defined as minimal disease activity (MDA) and Disease Activity in PsA (DAPSA) low disease activity (LDA).

**Results:**

A total of 1119 patients were classified into 5 distinct PsA phenotypes: Moderate to High Disease Activity (40.3% of patients) - characterized by lower tender joint count (TJC) and swollen joint count (SJC), dactylitis, and enthesitis; Enthesitis and Large Joints Dominant (20.8% of patients) - characterized by enthesitis and mainly active large joints; Very High Disease Activity (14.0% of patients) - characterized by high TJC/SJC, dactylitis, and enthesitis; Hand Dominant (13.8% of patients) - characterized by active joints primarily in the hands; Dactylitis and Feet Dominant (11.1% of patients) - characterized by dactylitis and active joints primarily in the feet. At 4 years, risankizumab demonstrated efficacy across all phenotypes (MDA and DAPSA LDA; range: 42.9% to 58.8% of patients and 66.0% to 82.0% of patients, respectively), with highest responses observed in the Moderate to High Disease Activity and Dactylitis and Feet Dominant phenotypes.

**Conclusions:**

Five distinct PsA phenotypes were identified in patients starting risankizumab. Moderate to High Disease Activity, the most frequent phenotype, showed the highest response, though risankizumab demonstrated efficacy across all phenotypes. These results are a first step toward more personalized medicine for patients with PsA.

**Trial registration:**

ClinicalTrials.gov: KEEPsAKE 1, NCT03675308; KEEPsAKE 2, NCT03671148.

**Supplementary Information:**

The online version contains supplementary material available at 10.1186/s13075-025-03670-0.

## Background

 Psoriatic arthritis (PsA) is an immune-mediated inflammatory rheumatic disease with heterogenous manifestations, including both musculoskeletal (peripheral arthritis, enthesitis, dactylitis, and/or axial involvement) and non-musculoskeletal (nail and skin psoriasis, as well as potentially uveitis and inflammatory bowel disease) domains. In a real-world cohort of patients with PsA in the US, most (~ 65%) were found to have a multidomain (≥ 2 domains) disease presentation versus single domain, which was associated with worse disease activity, quality of life (including pain and fatigue), and work productivity measures [1]. Due to the heterogeneity of PsA, disease domain type, frequency, and severity vary from patient-to-patient, and can impact treatment effectiveness and outcomes [1–3]. Therefore, a more personalized approach to medicine is needed to identify groups of patients with distinct clinical characteristics. It is possible that these unique phenotypes will respond differently to treatments, which would enable clinicians to select the most effective treatment that would result in the best clinical outcomes for patients with PsA [4].

Cluster analysis is a data-driven hypothesis-free unsupervised machine learning approach that can be used to identify clusters of patient phenotypes [5, 6]. Unsupervised learning algorithms, which do not rely on specific outcomes to help identify subgroups within datasets, can cluster patients into groups based on specified features to find homogeneous sub-phenotypes within a complex disease. These techniques can be used to better understand the etiology and pathophysiology of a disease, as well as provide a foundation for more targeted treatment to improve patient care [7]. In recent years, this approach has been utilized within the field of rheumatology [8], including in patients with systemic lupus erythematosus or PsA [9–11].

Risankizumab, an interleukin (IL)−23 p19 inhibitor, has demonstrated long-term safety and efficacy in adults with active PsA through 100 weeks of treatment in the phase 3 KEEPsAKE 1 and KEEPsAKE 2 trials [12–15]. The objectives of this post hoc analysis were to (1) classify patients into distinct PsA phenotypes based on baseline demographics and clinical characteristics using a machine learning approach, and (2) assess efficacy responses across PsA phenotypes in patients treated with risankizumab for up to 4 years (196 weeks) in the KEEPsAKE studies.

## Methods

### Patients and study design

The full methodological details for the KEEPsAKE 1 (NCT03675308) and KEEPsAKE 2 (NCT03671148) randomized controlled trials have been previously published [12, 13]. In brief, adult (≥ 18 years of age) patients with a confirmed clinical diagnosis of PsA were evaluated. For both studies, patients were required to have ≥ 5 tender joints based on 68 joint counts with ≥ 5 swollen joints based on 66 joint counts. Patients had an inadequate response or intolerance to ≥ 1 conventional synthetic disease-modifying antirheumatic drug (csDMARD) and some had an inadequate response or intolerance to 1 or 2 biologic DMARDs (bDMARDs). To ensure a more homogeneous patient cohort, only data from bDMARD-naïve patients were included in this post hoc analysis. The placebo-controlled period lasted 24 weeks in both studies, followed by an open-label period where all patients received risankizumab 150 mg every 12 weeks through week 316 in the ongoing long-term extension.

### Machine learning approach

#### Unsupervised machine learning model

Unsupervised machine learning, finite mixture model, was used to determine PsA phenotypic clusters, with baseline demographics and clinical characteristics as input features. Finite mixture models are probabilistic models that involve the combination of 2 or more density functions, which represent different subgroups (also known as latent classes) within an overall population [16]. In a finite mixture model, the observed responses $\:y$ are assumed to originate from $\:K$ distinct latent classes $\:{f}_{1},\:{f}_{2},\:\dots\:,\:{f}_{K}$ with proportions $\:{\pi\:}_{1},\:{\pi\:}_{2},\dots\:,\:{\pi\:}_{K}$, respectively. Formally, finite mixture model with $\:K$ components can be written as follows:

1 $$\:h\left(y\vert x,\:\phi\:\right)={\sum\:}_{k=1}^K{\pi\:}_kf_k\left(y\vert x,\:{\theta\:}_k\right)$$

where $\:y$ (could be multivariate) is a dependent variable with conditional density $\:h$, $\:x$ is a vector of independent variables, $\:{\pi\:}_{k}$ is the prior probability of component $\:k$, $\:{\theta\:}_{k}$ is the component specific parameter vector for the density function $\:{f}_{k}$, and $\phi=\left({\pi\:}_1,{\pi\:}_2,\dots\:,\:{\pi\:}_K,\:{\theta\:}_1,\:{\theta\:}_2,\:\dots\:,\:{\theta\:}_K\right)$ is the vector of all parameters. For the cluster analysis on PsA patients utilized here, $\:K$ is the total number of clusters identified among all the patients. Dependent variable $\:y$ is the observed baseline feature vector representing the baseline demographics and clinical characteristics of the patients. $\:{f}_{k}(\bullet\:)$ is the conditional probability density function for the baseline feature vector in the $\:k$ cluster. Independent variables (i.e., $\:x$) were not considered for this analysis. Therefore, the equation was simplified as follows:

2 $$\:\:\:\:\:\:\:\:\:\:\:\:\:\:\:\:\:\:\:\:\:\:\:\:\:\:h\left(y\vert\phi\:\right)=\:{\sum\:}_{k=1}^K{\pi\:}_kf_k\left(y\vert{\theta\:}_k\right)\:\:\:\:\:\:\:\:\:\:\:\:\:\:\:\:\:\:\:\:\:\:\:\:\:\:\:\:\:\:\:\:\:\:\:\:\:\:\:\:\:\:\:\:\:\:\:\:\:\:\:\:\:\:\:\:\:\:\:\:\:\:\:\:\:\:\:\:\:\:\:$$

#### Model fitting

An unsupervised machine learning model, finite mixture model, was applied to the combined dataset with both KEEPsAKE 1 and KEEPsAKE 2 study data. Baseline clinical characteristics were categorized into 6 groups: tender joint-related, swollen joint-related, psoriasis-related, dactylitis-related, enthesitis-related, and others. Race was coded as White, Black/African American, Asian, and multiple/others. Age was coded as ≤ 45, 45 to 65, and ≥ 65. Variables with excess missing data or high collinearity were excluded from the analysis, including 1 variable due to high missing data (> 20%) and 3 variables due to collinearity (Supplemental Table 1). Patients were removed from the dataset if they had missing data for any of the final set of feature variables. The detailed information for the final set of feature variables included in the cluster analysis can be found in Supplemental Table 2.

The Bayesian Information Criteria (BIC) was used to determine the number of clusters for the final model based on the grid search on a varying number of clusters (2 to 10) [17]. The optimal number of phenotypic clusters was determined using the BIC score, where a lower BIC score indicates higher model fitness while accounting for the model overfitting by penalizing models with more parameters. We selected 5 as the optimal number of clusters because the decrease of BIC is minimal when $\:k>5$ (Supplemental Fig. 1).

#### Evaluation of clustering results

To evaluate if the clustering results were clinically meaningful, the cluster results were visualized using a heatmap. Patients were ordered by cluster membership and the features by categories in the heatmap.

To assess the stability of the clustering results, The Monti consensus clustering algorithm was utilized. The Monti consensus clustering algorithm initially gained popularity in genomics [18], where a new molecular disease subtype was discovered [19, 20]. The algorithm generates a consensus matrix indicating the stability of clustering. Creation of the consensus matrix involves several steps: (1) randomly sample $\:a\%$ from the original data set $\:D$ to obtain the bootstrapped dataset $\:{D}_{t}$, where $\:t$ represents the $\:t$-th iteration, (2) for each random sampled dataset $\:{D}_{t}$, run cluster analysis with a pre-specified number of clusters, (3) repeat this process $\:T$ times, and (4) each time, generate an indicator matrix $\:{I}_{t}$ representing if 2 datapoints (e.g., $\:i,\:j$) are both selected in $\:{D}_{t}\:$and a cluster matrix $\:{M}_{t}$ representing if 2 data points (e.g., $\:i,\:j$) are in the same cluster. The consensus matrix $\:C$ can then be calculated as the fraction of times 2 samples are clustered together across $\:T$ iterations $\:\left(t=\text{1,2},\dots\:,\:T\right),$ where 2 samples are both selected in bootstrapped datasets as the following:

$$\:C\left(i,\:j\:\right)=\frac{{\sum\:}_{t=1}^{T}{M}^{t}(i,j)}{{\sum\:}_{t=1}^{T}{I}^{t}(i,j)}$$

For this post hoc analysis, this process was repeated 200 times to generate the consensus matrix to evaluate model stability [11]. Each time, 80% of the data were sampled for clustering. Patients were ordered by cluster membership and represented visually in a matrix (Supplemental Fig. 2).

Statistical software R (version 4.1.2) was used for data processing and statistical analysis. The unsupervised machine learning model was performed using the R ’fpc’ and ’flexmix’ package [21].

### Outcomes and statistical analysis

Baseline demographics and clinical characteristics for each PsA phenotype are described. In addition, efficacy responses across the PsA phenotypes were assessed at weeks 12, 24, 52, 100, and 196 in patients who received continuous risankizumab 150 mg treatment. Efficacy endpoints included the proportions of patients who achieved minimal disease activity (MDA), Disease Activity in PsA (DAPSA) low disease activity (LDA; score ≤ 14), tender joint count (TJC) improvement from baseline ≥ 50%, swollen joint count (SJC) improvement from baseline ≥ 50%, and ≥ 50% improvement in the American College of Rheumatology response criteria (ACR50), as well as minimal clinically important difference (MCID) in the Health Assessment Questionnaire - Disability Index (HAQ-DI; score decrease of ≥ 0.35), MCID in the patient’s assessment of pain (score decrease of visual analog scale [VAS] ≥ 10 mm), and MCID in the Functional Assessment of Chronic Illness Therapy – Fatigue (FACIT-F; ≥ 4 increase from baseline). The safety of risankizumab was not assessed in this post hoc analysis; however, risankizumab was well tolerated and no new safety signals were observed in the KEEPsAKE primaries and open-label long-term extensions compared with previous reports [12–15].

Descriptive statistics were used to summarize baseline demographic and clinical characteristic data for each PsA phenotype. Efficacy endpoints were analyzed using as observed or non-responder imputation incorporating multiple imputation (NRI-MI) for those missing data due to COVID-19 or geopolitical conflict in Ukraine, Russia, or Israel based on as observed data.

## Results

### Identification of PsA phenotypes

**Fig. 1:**
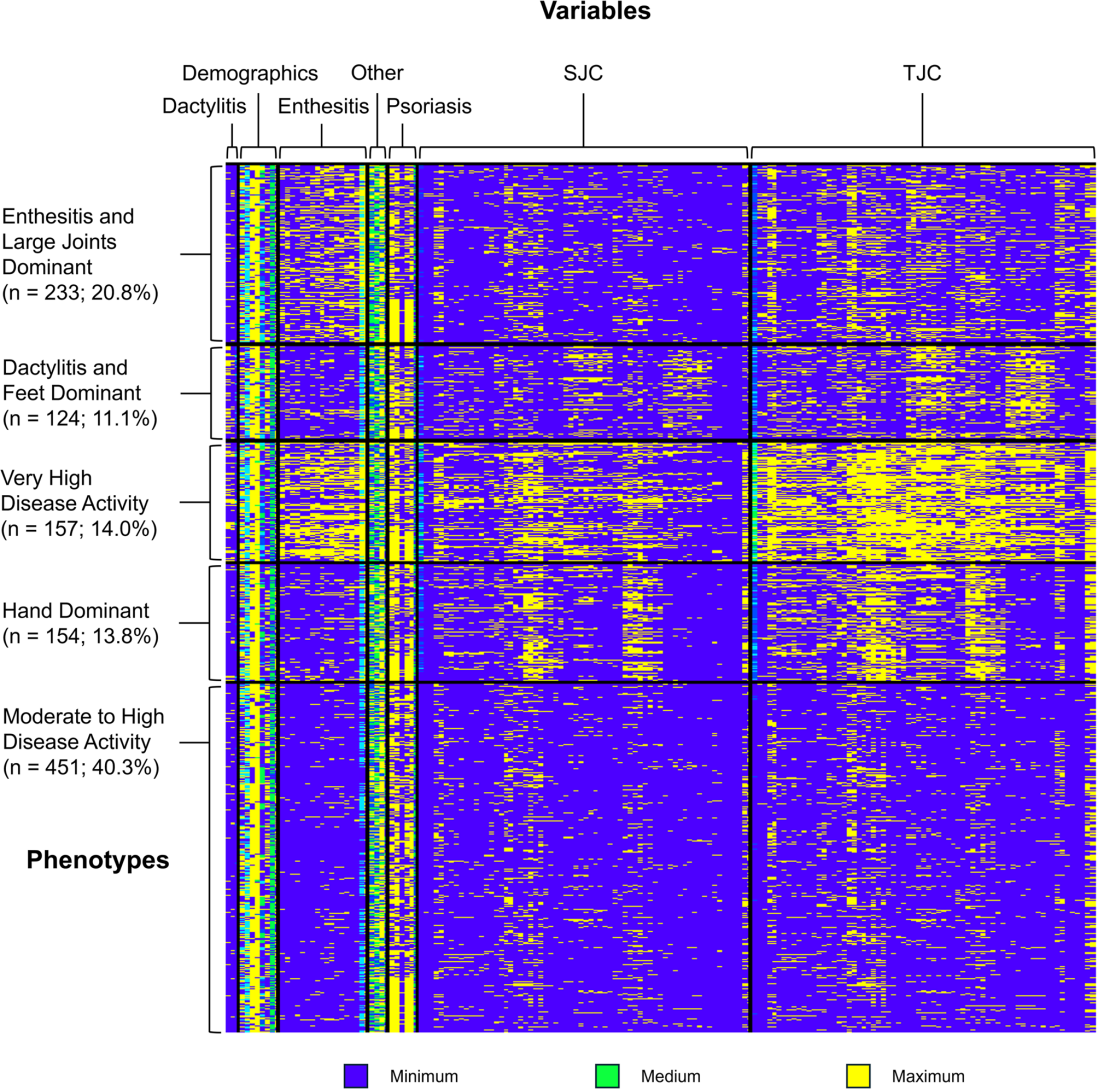
Identified PsA Phenotypes Based on Frequency of Baseline Demographics and Clinical Characteristics. After exclusions, variables were defined as follows: dactylitis (number of joints affected by dactylitis, presence of dactylitis in hands, presence of dactylitis in feet), demographics (age, sex, BMI, smoking status, race, ethnicity, geographic region, work status), enthesitis (presence of enthesitis, SPARCC index score, location[s] of enthesitis), other (pain visual analog scale, FACIT-F score, CRP level, duration of PsA), psoriasis (skin involvement, location affected by psoriasis [head, lower extremities, trunk, upper extremities], psoriatic spondylitis), SJC (swollen joint involvement [by joint, yes/no], SJC66 [swollen joint count in 66 joints]), and TJC (tender joint involvement [by joint, yes/no], TJC68 [tender joint count in 68 joints]). *BMI* body mass index, *CRP* C-reactive protein, *FACIT-F* Functional Assessment of Chronic Illness Therapy - Fatigue, *PsA* psoriatic arthritis, *SJC* swollen joint count, *SPARCC* Spondyloarthritis Research Consortium of Canada, *TJC* tender joint count

**Fig. 2:**
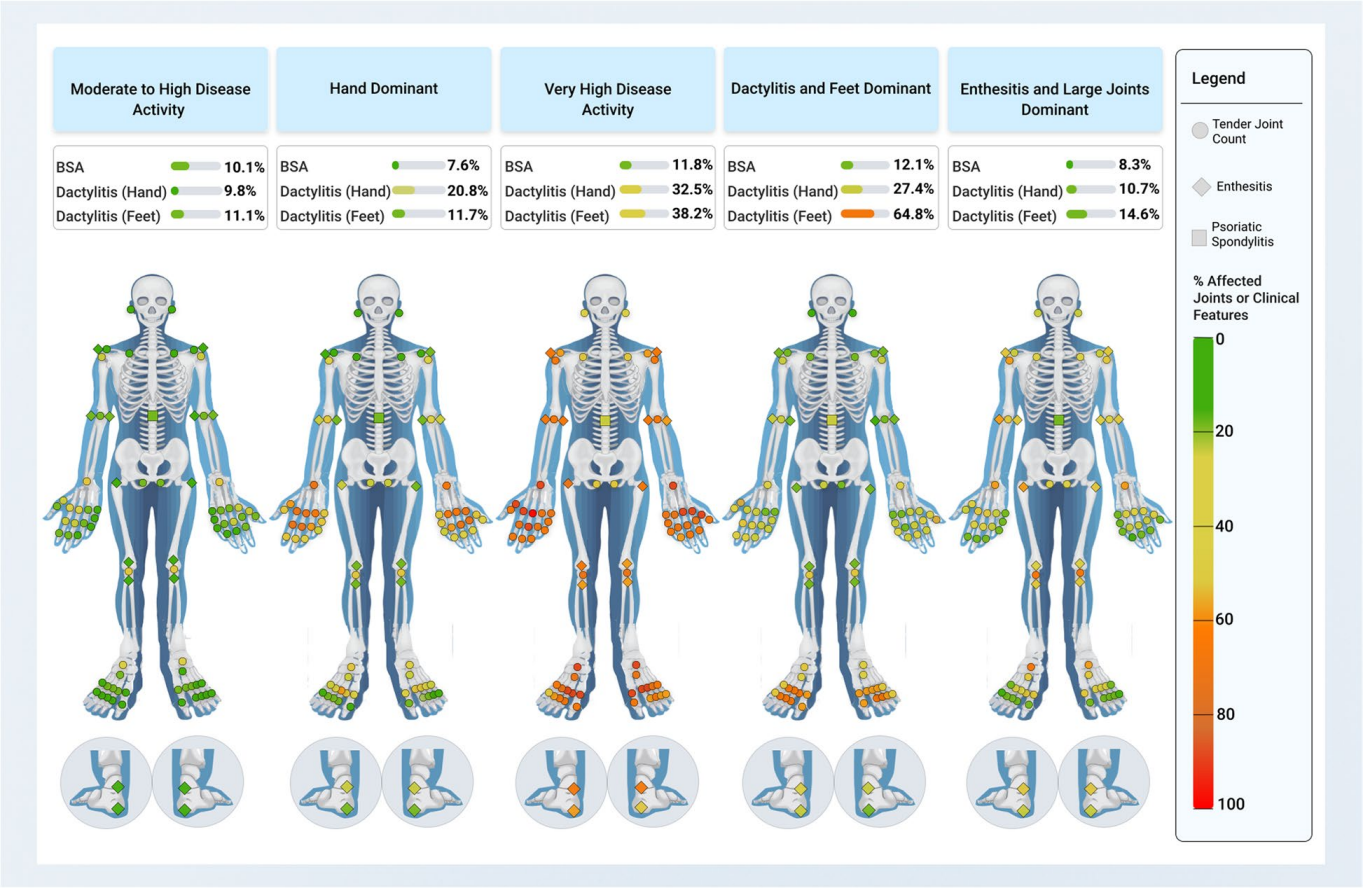
Schematic of Identified PsA Phenotypes. PsA phenotypes identified using an unsupervised machine learning approach based on baseline demographics and clinical characteristics of PsA. *BSA* body surface area, *PsA* psoriatic arthritis

Of 1196 patients, after excluding those with missing data, 1119 (93.6%) bDMARD-naïve patients with PsA were included in this post hoc analysis. Using cluster analysis (Fig. 1), 5 distinct PsA phenotypes were identified based on the frequency of baseline demographics and clinical characteristics (Fig. 2). Baseline demographics and clinical characteristics for the 5 PsA phenotypes are shown in Table 1.

**Table 1:** Patient Baseline Demographics and Clinical Characteristics Stratified by PsA Phenotype^a^

Parameter	Moderate to High Disease Activity 451 (40.3)	Enthesitis and Large Joints Dominant 233 (20.8)	Very High Disease Activity 157 (14.0)	Hand Dominant 154 (13.8)	Dactylitis and Feet Dominant 124 (11.1)
Age (years), n (%)					
≤ 45	176 (39.0)	73 (31.3)	35 (22.3)	46 (29.9)	57 (46.0)
>45 to ≤ 65	220 (48.8)	124 (53.2)	100 (63.7)	84 (54.5)	62 (50.0)
>65	55 (12.2)	36 (15.5)	22 (14.0)	24 (15.6)	5 (4.0)
Age (years), mean (SD)	49.9 (12.3)	51.5 (11.9)	53.6 (11.1)	53.2 (12.0)	47.5 (11.3)
Sex, n (%)					
Female	200 (44.3)	145 (62.2)	94 (59.9)	84 (54.5)	45 (36.3)
Male	251 (55.7)	88 (37.8)	63 (40.1)	70 (45.5)	79 (63.7)
BMI (kg/m^2^), n (%)					
< 25	84 (18.6)	33 (14.2)	24 (15.3)	22 (14.3)	32 (25.8)
≥ 25 to < 30	184 (40.8)	66 (28.3)	45 (28.7)	68 (44.2)	43 (34.7)
≥ 30	183 (40.6)	134 (57.5)	88 (56.1)	64 (41.6)	49 (39.5)
BMI (kg/m^2^), mean (SD)	29.8 (6.1)	32.2 (7.7)	32.0 (7.0)	30.1 (5.4)	29.4 (6.2)
PsA disease duration (years), n (%)					
< 2	136 (30.2)	59 (25.3)	42 (26.8)	38 (24.7)	29 (23.4)
2 to 5	120 (26.6)	71 (30.5)	36 (22.9)	37 (24.0)	43 (34.7)
5 to 10	104 (23.1)	57 (24.5)	33 (21.0)	39 (25.3)	27 (21.8)
≥ 10	91 (20.2)	46 (19.7)	46 (29.3)	40 (26.0)	25 (20.2)
PsA disease duration (years), mean (SD)	6.3 (6.7)	6.8 (6.9)	7.8 (7.8)	7.8 (8.1)	6.1 (5.8)
CRP, n (%)					
< 1	55 (12.2)	15 (6.4)	17 (10.8)	10 (6.5)	5 (4.0)
1 to < 3	84 (18.6)	60 (25.8)	30 (19.1)	41 (26.6)	28 (22.6)
3 to < 10	194 (43.0)	93 (39.9)	69 (43.9)	64 (41.6)	44 (35.5)
≥ 10	118 (26.2)	65 (27.9)	41 (26.1)	39 (25.3)	47 (37.9)
TJC, mean (SD)	10.1 (3.3)	20.0 (7.2)	46.6 (9.8)	25.3 (7.0)	24.7 (8.4)
SJC, mean (SD)	7.4 (2.1)	10.1 (4.0)	24.0 (11.3)	15.0 (5.5)	15.5 (7.9)
DAPSA score, mean (SD)	29.2 (6.8)	42.8 (10.5)	84.8 (16.1)	53.2 (10.9)	52.5 (14.8)
Dactylitis (LDI > 0), n (%)	83 (18.4)	49 (21.0)	74 (47.1)	41 (26.6)	75 (60.5)
Number of joints affected by dactylitis,^b^ mean (SD)	1.5 (0.8)	1.5 (0.8)	4.0 (2.9)	2.1 (1.2)	2.8 (1.6)
Enthesitis score, n (%)					
0	244 (54.1)	3 (1.3)	7 (4.5)	44 (28.6)	29 (23.4)
1 to 5	203 (45.0)	73 (31.3)	22 (14.0)	81 (52.6)	76 (61.3)
6 to 16	4 (0.9)	157 (67.4)	128 (81.5)	29 (18.8)	19 (15.3)
Enthesitis score,^c^ mean (SD)	2.3 (1.2)	6.9 (3.0)	10.0 (3.7)	4.0 (2.4)	3.9 (2.3)
Psoriatic spondylitis,^d^ n (%)	82 (18.2)	39 (16.7)	35 (22.3)	22 (14.3)	36 (29.0)
Skin involvement (% BSA), n (%)					
0	20 (4.4)	14 (6.0)	5 (3.2)	8 (5.2)	9 (7.3)
< 3	170 (37.7)	107 (45.9)	65 (41.4)	54 (35.1)	39 (31.5)
≥ 3 to ≤ 10	155 (34.4)	64 (27.5)	43 (27.4)	56 (36.4)	39 (31.5)
≥ 10	106 (23.5)	48 (20.6)	44 (28.0)	36 (23.4)	37 (29.8)
Skin involvement (% BSA),^e^ mean (SD)	10.1 (16.9)	8.3 (15.7)	11.8 (18.4)	7.6 (11.1)	12.1 (19.6)
Pain (0 to 100 VAS), n (%)					
≤ 15	34 (7.5)	7 (3.0)	1 (0.6)	3 (1.9)	7 (5.6)
>15 to ≤ 30	51 (11.3)	19 (8.2)	8 (5.1)	13 (8.4)	16 (12.9)
>30 to < 80	319 (70.7)	170 (73.0)	105 (66.9)	115 (74.7)	83 (66.9)
≥ 80	47 (10.4)	37 (15.9)	43 (27.4)	23 (14.9)	18 (14.5)
Pain (0 to 100 VAS), mean (SD)	52.6 (23.0)	57.7 (21.6)	66.5 (19.7)	59.0 (20.3)	54.8 (23.9)

*BMI* body mass index, BSA body surface area, *CRP* C-reactive protein, *DAPSA* Disease Activity in Psoriatic Arthritis, *LDI* Leeds Dactylitis Index, *PsA* psoriatic arthritis, *SJC* swollen joint count, *SPARCC* Spondyloarthritis Research Consortium of Canada, *TJC* tender joint count, *VAS* visual analog scale

^a^Baseline data for each PsA phenotype include patients who received placebo or risankizumab 150 mg

^b^For patients with LDI > 0 at baseline

^c^Mean SPARCC enthesitis index for patients with SPARCC > 0 at baseline

^d^Psoriatic spondylitis was determined by the clinical trial investigator at baseline, which may have included radiographic data; details regarding how a diagnosis was determined (i.e., clinical evaluation, radiography, and/or MRI) were recorded

^e^For patients with BSA > 0 at baseline

#### Moderate to high disease activity

The most frequent phenotypic cluster was Moderate to High Disease Activity (*n* = 451; 40.3% of patients). At baseline, patients in this phenotype had the lowest mean TJC (10.1) and SJC (7.4) values, the lowest proportion of patients with dactylitis (18.4%), and the lowest mean enthesitis score (2.3) within the KEEPsAKE clinical trial population. Additionally, these patients tended to be slightly younger (mean age: 49.9 years old), were closely split amongst the sexes (44.3% female, 55.7% male), and a higher proportion had a body mass index (BMI) < 25 (18.6%) compared with most other phenotypes. Patients in this phenotype had one of the shortest mean disease durations (6.3 years) and the lowest mean DAPSA score (29.2). The large majority of patients had skin involvement (total 95.6%).

#### Enthesitis and large joints dominant

The second most frequent phenotypic cluster was Enthesitis and Large Joints Dominant (*n* = 233; 20.8% of patients). At baseline, patients in this phenotype had the second highest mean enthesitis score (6.9), as well as moderate mean TJC (20.0) and SJC (10.1) values, with mainly active large joints. This phenotype had the highest proportion of females (62.2%) and the highest proportion of patients with a BMI ≥ 30 (57.5%) compared with the other phenotypes. Most patients had skin involvement (total 94.0%).

#### Very high disease activity

The third most frequent phenotypic cluster was Very High Disease Activity (*n* = 157; 14.0% of patients). At baseline, patients in this phenotype had the highest mean TJC (46.6) and SJC (24.0) values, as well as a higher proportion of patients with dactylitis (47.1%) and the highest mean enthesitis score (10.0). These patients were the oldest (mean age: 53.6 years old), a higher proportion were female (59.9%), and a higher proportion had a BMI ≥ 30 (56.1%) compared with most other phenotypes. These patients had the longest mean disease duration (7.8 years), which was the same for the Hand Dominant phenotype (described below). Additionally, these patients had the highest mean DAPSA score (84.8) and the highest mean pain score (66.5). Similar to the other phenotypes, the large majority of patients had skin involvement (total 96.8%).

#### Hand dominant

The fourth most frequent phenotypic cluster was Hand Dominant (*n* = 154; 13.8% of patients). At baseline, patients in this phenotype had moderate mean TJC (25.3) and SJC (15.0) values, with active joints primarily in the hands. These patients tended to be older (mean age: 53.2 years old) and were closely split amongst the sexes (54.5% female, 45.5% male). Additionally, as stated above, these patients had the longest mean disease duration (7.8 years), which was the same for the Very High Disease Activity phenotype. As was common to the other phenotypes, most patients had skin involvement (total 94.8%).

#### Dactylitis and feet dominant

The fifth and least frequent phenotypic cluster was Dactylitis and Feet Dominant (*n* = 124; 11.1% of patients). At baseline, patients in this phenotype had the highest proportion of patients with dactylitis (60.5%), as well as higher mean TJC (24.7) and SJC (15.5) values. In contrast to the Hand Dominant phenotype, the active joints were primarily in the feet. These patients were the youngest (mean age: 47.5 years old), the highest proportion were male (63.7%), and the highest proportion with a BMI < 25 (25.8%) compared with the other phenotypes. Furthermore, these patients had the shortest mean disease duration (6.1 years), the highest proportion of patients with psoriatic spondylitis (29.0%), and most had skin involvement (total 92.7%).

### Efficacy endpoints by PsA phenotypic clusters

**Fig. 3:**
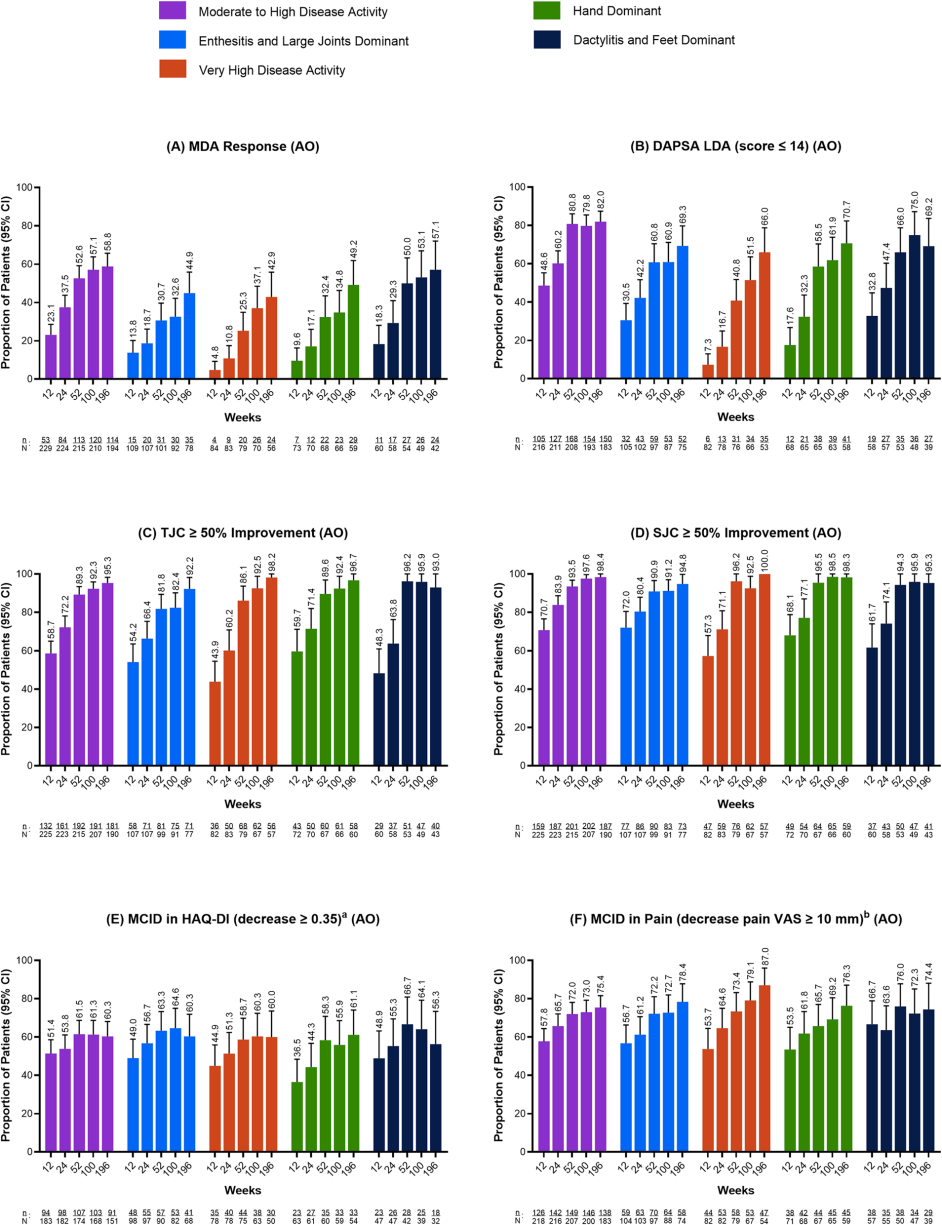
Efficacy Responses Across Psa Phenotypes Among BDMARD-Naïve Patients Receiving Continuous Risankizumab 150 mg (As Observed). Efficacy endpoints were analyzed using as observed data. ^a^Analysis in patients with baseline HAQ-DI ≥ 0.35. ^b^Analysis in patients with baseline pain VAS ≥ 10 mm. *AO *as observed, *bDMARD *biologic Disease-Modifying Antirheumatic Drug, CI confidence interval, *DAPSA *Disease Activity in Psoriatic Arthritis, *HAQ-DI* Health Assessment Questionnaire – Disability Index, *LDA *low disease activity, *MCID *minimal clinically important difference, *MDA *minimal disease activity, *PsA *psoriatic arthritis, *SJC *swollen joint count, *TJC *tender joint count, *VAS *visual analog scale

Overall, risankizumab demonstrated efficacy across several measures of disease activity through 4 years (196 weeks) of treatment across all PsA phenotypes (Fig. 3). While patients in all phenotypes demonstrated improvement up to 4 years, the proportion of patients reaching MDA and LDA targets did vary by phenotypic cluster. Achievement of MDA varied across phenotypes, with the highest proportion of patients observed in the Moderate to High Disease Activity phenotype and the lowest in the Very High Disease Activity phenotype at all time points assessed (weeks 12, 24, 52, 100, and 196) (Fig. 3A). At week 196, 58.8% of patients in the Moderate to High Disease Activity phenotype achieved MDA response versus 42.9% of patients in the Very High Disease Activity phenotype. Similarly, achievement of DAPSA LDA varied across phenotypes, with the highest responses observed in the Moderate to High Disease Activity phenotype and the lowest in the Very High Disease Activity phenotype through week 196 (Fig. 3B). At week 196, 82.0% of patients achieved DAPSA LDA in the Moderate to High Disease Activity phenotype versus 66.0% in the Very High Disease Activity phenotype.

The proportion of patients achieving TJC ≥ 50% improvement from baseline and SJC ≥ 50% improvement from baseline were largely similar across the phenotypes, with 92.2% to 98.2% of patients achieving ≥ 50% improvement in TJC and 94.8% to 100.0% achieving ≥ 50% improvement in SJC, at week 196 (as observed: Figure 3C and D). Achievement of ACR50 varied across phenotypes, with the highest responses observed in the Very High Disease Activity (57.4%) and Dactylitis and Feet Dominant (59.0%) phenotypes, at week 196 (as observed: Supplemental Fig. 3A).

The proportion of patients achieving MCID in HAQ-DI were largely similar across the PsA phenotypes, with the highest response observed in the Hand Dominant phenotype (61.1%) and lowest response observed in the Dactylitis and Feet Dominant phenotype (56.3%), at week 196 (as observed: Fig. 3E). At week 196, the proportion of patients achieving MCID in pain was generally similar across the PsA phenotypes (range: 74.4% to 78.4%), with the exception of the Very High Disease Activity phenotype, which showed 87.0% of patients achieving a response (as observed: Fig. 3F). Achievement of MCID in FACIT-F varied across the phenotypes, with the highest proportion of patients observed in the Very High Disease Activity phenotype (78.2%) and the lowest proportion of patients observed in the Moderate to High Disease Activity phenotype (58.1%), at week 196 (as observed: Supplemental Fig. 3B).

Within each phenotype, efficacy responses were generally maintained, and in many cases further improved, from week 12 through week 196 of risankizumab treatment for all endpoints evaluated (as observed: Fig. 3 and Supplemental Fig. 3). Across the efficacy endpoints, more conservative estimates using NRI-MI showed generally similar results to the as observed analyses (Supplemental Fig. 4 and Supplemental Fig. 5).

## Discussion

In this post hoc analysis of the KEEPsAKE studies, a machine learning approach was used to identify 5 distinct PsA phenotypes based on baseline demographics and clinical characteristics: Moderate to High Disease Activity, Enthesitis and Large Joints Dominant, Very High Disease Activity, Hand Dominant, and Dactylitis and Feet Dominant. These 5 phenotypes showed considerable variation across baseline parameters, including age, sex, BMI, PsA disease duration, C-reactive protein, mean TJC and SJC, location of tender and swollen joints, DAPSA score, dactylitis and enthesitis, psoriatic spondylitis, skin involvement, and pain. Overall, patients in the Moderate to High Disease Activity phenotype tended to have lower disease activity, representing a phenotype closer to those in clinical practice, while those in the Very High Disease Activity phenotype had the greatest disease activity, compared with the other phenotypes at baseline.

Across all PsA phenotypes, patients treated with risankizumab through 4 years (196 weeks) demonstrated efficacy across several measures of disease activity, including MDA and DAPSA LDA. Achievement of MDA and DAPSA LDA was highest in the Moderate to High Disease Activity phenotype, which is not surprising given the lower disease activity reported by these patients at baseline. In highly active patients with very high joint burden, a lower proportion of patients achieved efficacy endpoints, as it is more challenging to achieve disease control in this population. Within each phenotype, progressive improvements in response over time were observed for nearly all efficacy endpoints from weeks 12 to 196, demonstrating the sustained benefit of risankizumab across the diversity of PsA domains.

Unsupervised machine learning is a more recent application of artificial intelligence, which employs data-driven pattern detection (clustering) of patients without using predefined clinical criteria [9, 22, 23]. In rheumatology, this approach can be utilized to identify similar experiences within groups of patients, allowing for a more tailored/personalized treatment approach to optimize clinical outcomes [9]. Several recent post hoc analyses using clinical trial data from patients with PsA utilized a machine learning approach to identify 7 or 8 distinct PsA phenotypes [11, 24, 25], which included some overlap with the phenotypes described here (i.e., Hand Dominant, Enthesitis and Large Joints) [25]. As the use of machine learning expands, differences in phenotypic clustering are likely to occur due to variability in methodology, as well as the patient populations assessed. Altogether, these data demonstrate the application of a machine learning approach to clinical study data to identify distinct patient phenotypes that vary according to baseline demographics and clinical characteristics.

Several limitations of this analysis should be acknowledged. First, this analysis of patients from two phase 3 clinical trials is not fully representative of patients in clinical practice; further validation of these findings using real-world data are needed. This is supported by findings from a recent meta-analysis of patients with PsA, which found that those in randomized controlled trials appeared to have higher disease activity compared with those in real-world studies [26], which could potentially lead to fewer patients achieving treatment targets, such as MDA and DAPSA, in clinical trials. Second, both KEEPsAKE studies required that patients have ≥ 5 tender and ≥ 5 swollen joints to be included, which may have excluded patients with low joint involvement. Due to both of these limitations combined, there may be additional PsA phenotypes that were not identified in this analysis. Third, as is common to long-term open-label studies, patients who respond well to treatment via higher efficacy or lower adverse events are more likely to remain in the study. Therefore, data from as observed analyses should be interpreted with care, as they may overestimate treatment efficacy, especially at later timepoints. To combat this inherent bias in analyses of long-term data, more conservative estimates using NRI-MI analyses have been provided in the supplemental materials.

## Conclusions

In summary, a machine learning approach was utilized to identify 5 distinct PsA phenotypes using patient baseline demographics and clinical characteristics from the phase 3 KEEPsAKE studies. Risankizumab demonstrated efficacy across all 5 phenotypes through 4 years (196 weeks) of treatment across several measures of disease activity, including MDA and DAPSA LDA. While patients in all phenotypes demonstrated improvement up to 4 years, some variability was observed across phenotypes in the proportion of patients reaching MDA/LDA targets. A better understanding of PsA phenotypes could help to inform treatment decisions and improve patient care.

## Supplementary Information

Supplementary Material 1: Supplementary Material_Final

## Data Availability

AbbVie is committed to responsible data sharing regarding the clinical trials we sponsor. This includes access to anonymized, individual, and trial-level data (analysis data sets), as well as other information (e.g., protocols, clinical study reports, synopses, or analysis plans), as long as the trials are not part of an ongoing or planned regulatory submission. These clinical trial data can be requested by any qualified researchers who engage in rigorous, independent, scientific research, and will be provided following review and approval of a research proposal, Statistical Analysis Plan (SAP), and execution of a Data Use Agreement (DUA). Data requests can be submitted at any time after approval in the US and Europe and after acceptance of this manuscript for publication. The data will be accessible for 12 months, with possible extensions considered. For more information on the process or to submit a request, visit the following link: https://vivli.org/ourmember/abbvie/ then select “Home”.

## Abbreviations

ACR: American College of Rheumatology
bDMARD: biologic Disease-Modifying Antirheumatic Drug
BIC: Bayesian Information Criteria
csDMARD: Conventional Synthetic Disease-Modifying Antirheumatic Drug
DAPSA: Disease Activity in Psoriatic Arthritis
FACIT-F: Functional Assessment of Chronic Illness Therapy - Fatigue
HAQ-DI: Health Assessment Questionnaire - Disability Index
IL: Interleukin
LDA: Low Disease Activity
MCID: Minimal Clinically Important Difference
MDA: Minimal Disease Activity
PsA: Psoriatic Arthritis
SJC: Swollen Joint Count
TJC: Tender Joint Count
VAS: Visual Analog Scale
