# Supplementary material for: Efficacy of Risankizumab across distinct PsA phenotypes identified with machine learning analytics using data from biologic DMARD-Naïve patients in two phase 3 clinical trials
Source: Arthritis Res Ther. 2025 Nov 29;28:2. doi: 10.1186/s13075-025-03670-0 (PMC12771703; doi:10.1186/s13075-025-03670-0)
Supplement: Supplementary file 1 — Supplementary Material 1: Supplementary Material_Final [file 13075_2025_3670_MOESM1_ESM.docx]

**SUPPLEMENTAL MATERIALS**

**Efficacy of Risankizumab Across Distinct PsA Phenotypes Identified With Machine Learning Analytics Using Data From Biologic DMARD-Naïve Patients in Two Phase 3 Clinical Trials**

Laure Gossec, MD, PhD; Andra Balanescu, MD, PhD; Maria Antonietta D’Agostino, MD, PhD; Alexis Ogdie, MD; Philipp Sewerin, MD, PhD; Yu Deng, PhD; Linyu Shi, PhD; Yoshiyuki Sugimoto, PhD; Sheng Zhong, PhD; Yunzhao Xing, PhD; Ralph Lippe, PhD; Mitsumasa Kishimoto, MD, PhD

**Supplemental FigurES**

**Supplemental Figure 1. BIC Score to Determine Optimal Number of PsA Phenotypes**

The BIC score is plotted with varying number of clusters, ranging from 2 to 9. The BIC score was used to determine the optimal number of clusters for the final unsupervised machine learning model, where a lower BIC score indicates higher model fitness. The ‘elbow’ is indicated with a red circle on the figure and indicates the number of clusters that should be used. For clusters 6, 7, and 8, the machine learning algorithm did not converge, and therefore, no data points are shown for these clusters in the figure. BIC, Bayesian Information Criteria; PsA, psoriatic arthritis.

**
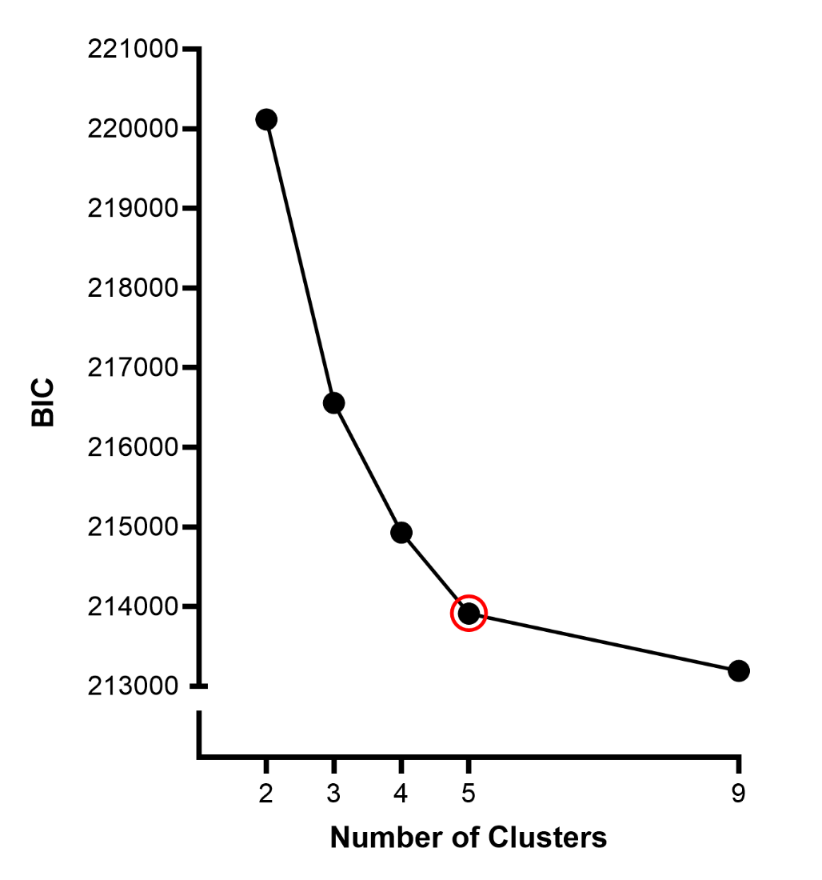
**

**Supplemental Figure 2. Consensus Matrix to Assess PsA Phenotype Cluster Stability**

Each entry $i, j$ represents the fraction of times 2 samples clustered together across 200 iterations, where 2 samples are both selected in bootstrapped datasets. PsA, psoriatic arthritis.


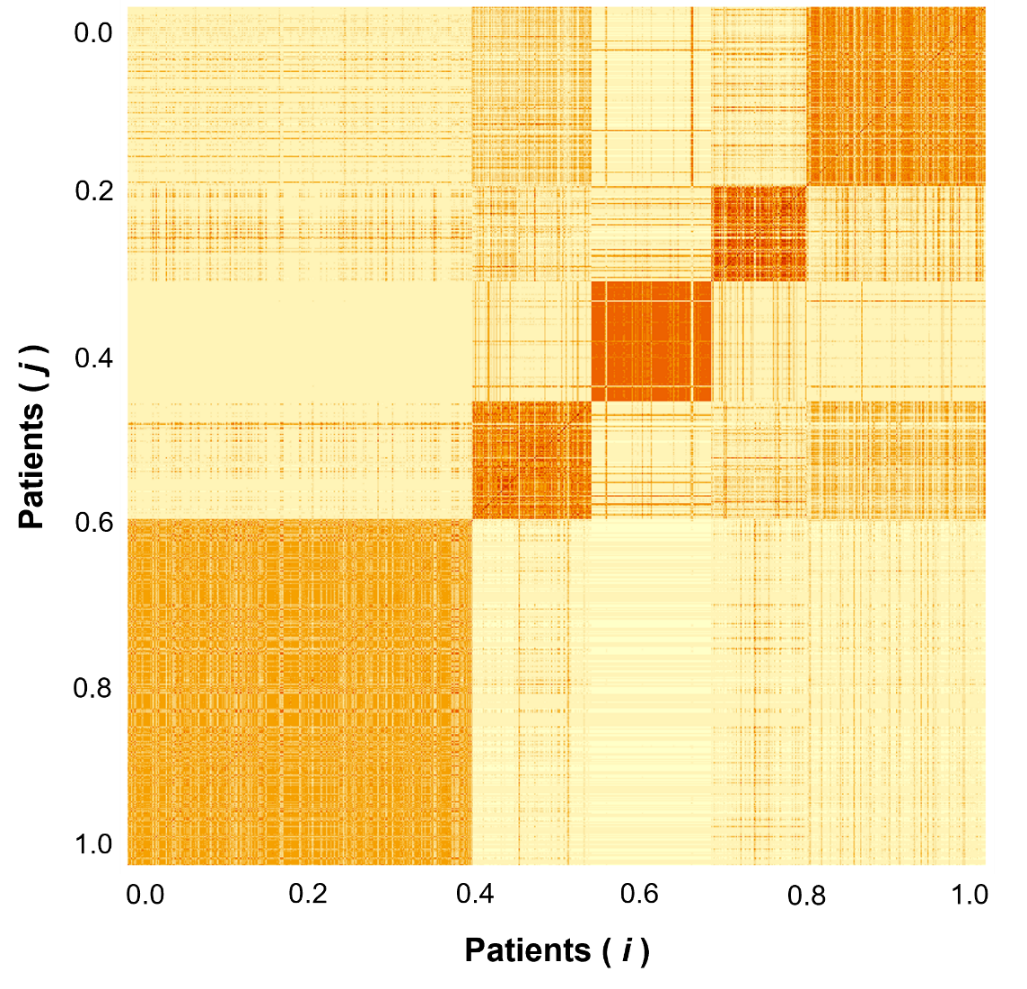


**Supplemental Figure 3. Additional Efficacy Responses Across PsA Phenotypes Among bDMARD-Naïve Patients Receiving Continuous Risankizumab 150 mg (As Observed)**

Efficacy endpoints were analyzed using as observed data. ACR50, ≥ 50% improvement in the American College of Rheumatology response criteria; AO, as observed; bDMARD, biologic Disease-Modifying Antirheumatic Drug; CI, confidence interval; FACIT-F, Functional Assessment of Chronic Illness Therapy - Fatigue; MCID, minimal clinically important difference; PsA, psoriatic arthritis.


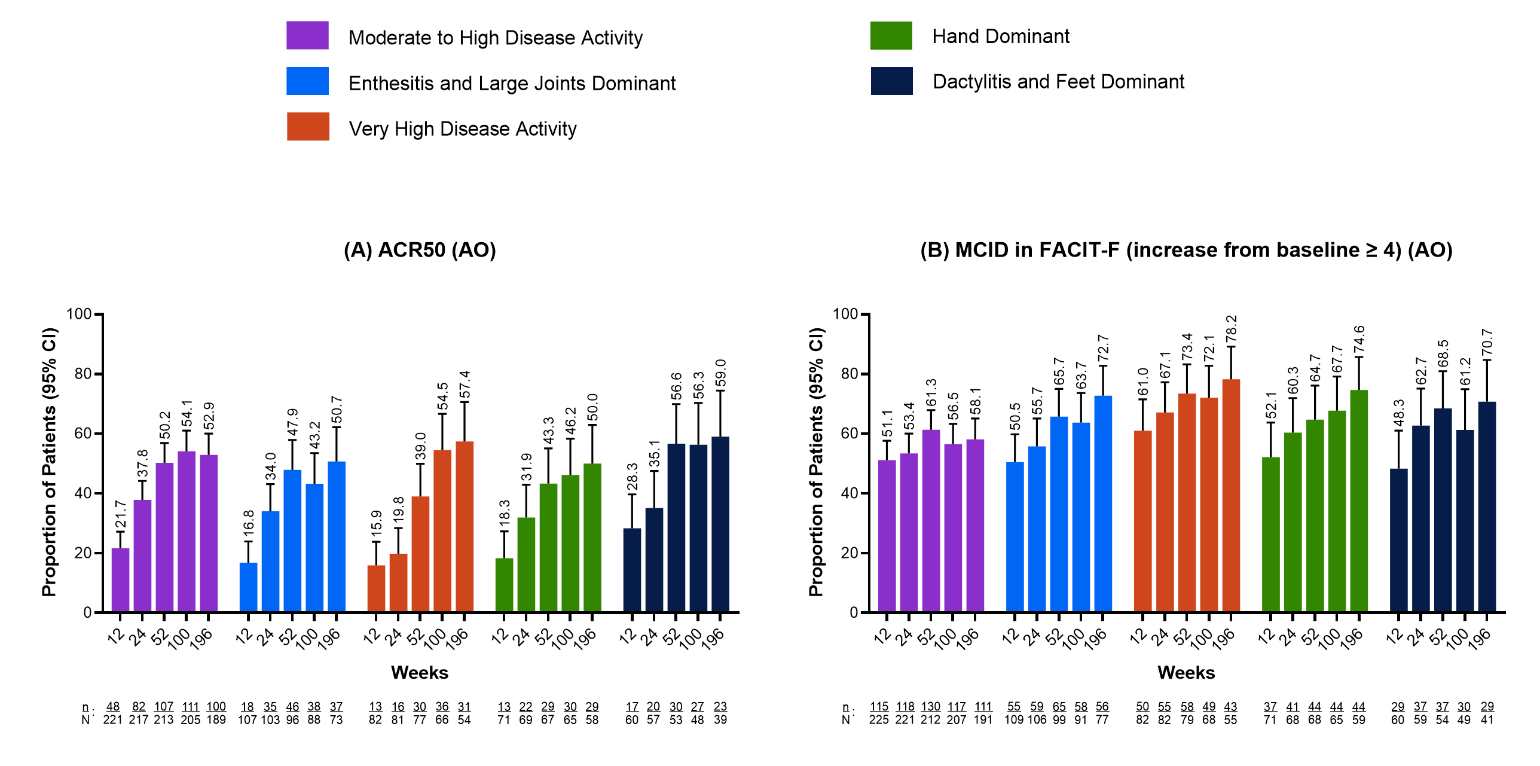


**Supplemental Figure 4. Efficacy Responses Across PsA Phenotypes Among bDMARD-Naïve Patients Receiving Continuous Risankizumab 150 mg (NRI-MI)**

Efficacy endpoints were analyzed using NRI-MI for those missing data due to COVID-19 or geopolitical conflict in Ukraine, Russia, or Israel based on as observed data. ^a^Analysis in patients with baseline HAQ-DI ≥ 0.35. ^b^Analysis in patients with baseline pain VAS ≥ 10 mm. bDMARD, biologic Disease-Modifying Antirheumatic Drug; CI, confidence interval; DAPSA, Disease Activity in Psoriatic Arthritis; HAQ-DI, Health Assessment Questionnaire – Disability Index; LDA, low disease activity; MCID, minimal clinically important difference; MDA, minimal disease activity; NRI-MI, nonresponder imputation incorporating multiple imputation; PsA, psoriatic arthritis; SJC, swollen joint count; TJC, tender joint count; VAS, visual analog scale.


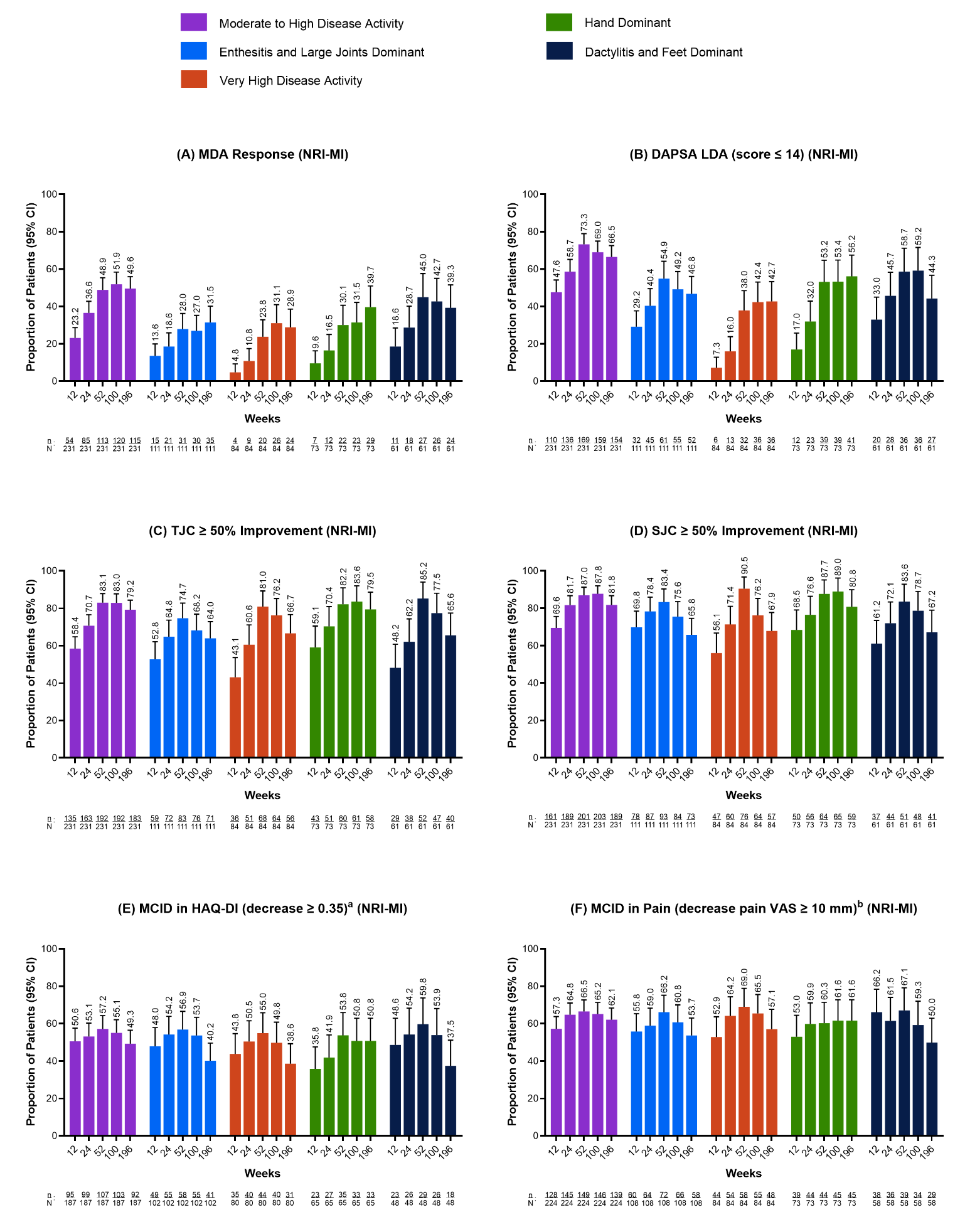


**Supplemental Figure 5. Additional Efficacy Responses Across PsA Phenotypes Among bDMARD-Naïve Patients Receiving Continuous Risankizumab 150 mg (NRI-MI)**

Efficacy endpoints were analyzed using NRI-MI for those missing data due to COVID-19 or geopolitical conflict in Ukraine, Russia, or Israel based on as observed data. ACR50, ≥ 50% improvement in the American College of Rheumatology response criteria; bDMARD, biologic Disease-Modifying Antirheumatic Drug; CI, confidence interval; FACIT-F, Functional Assessment of Chronic Illness Therapy – Fatigue; MCID, minimal clinically important difference; NRI-MI, nonresponder imputation incorporating multiple imputation; PsA, psoriatic arthritis.

**
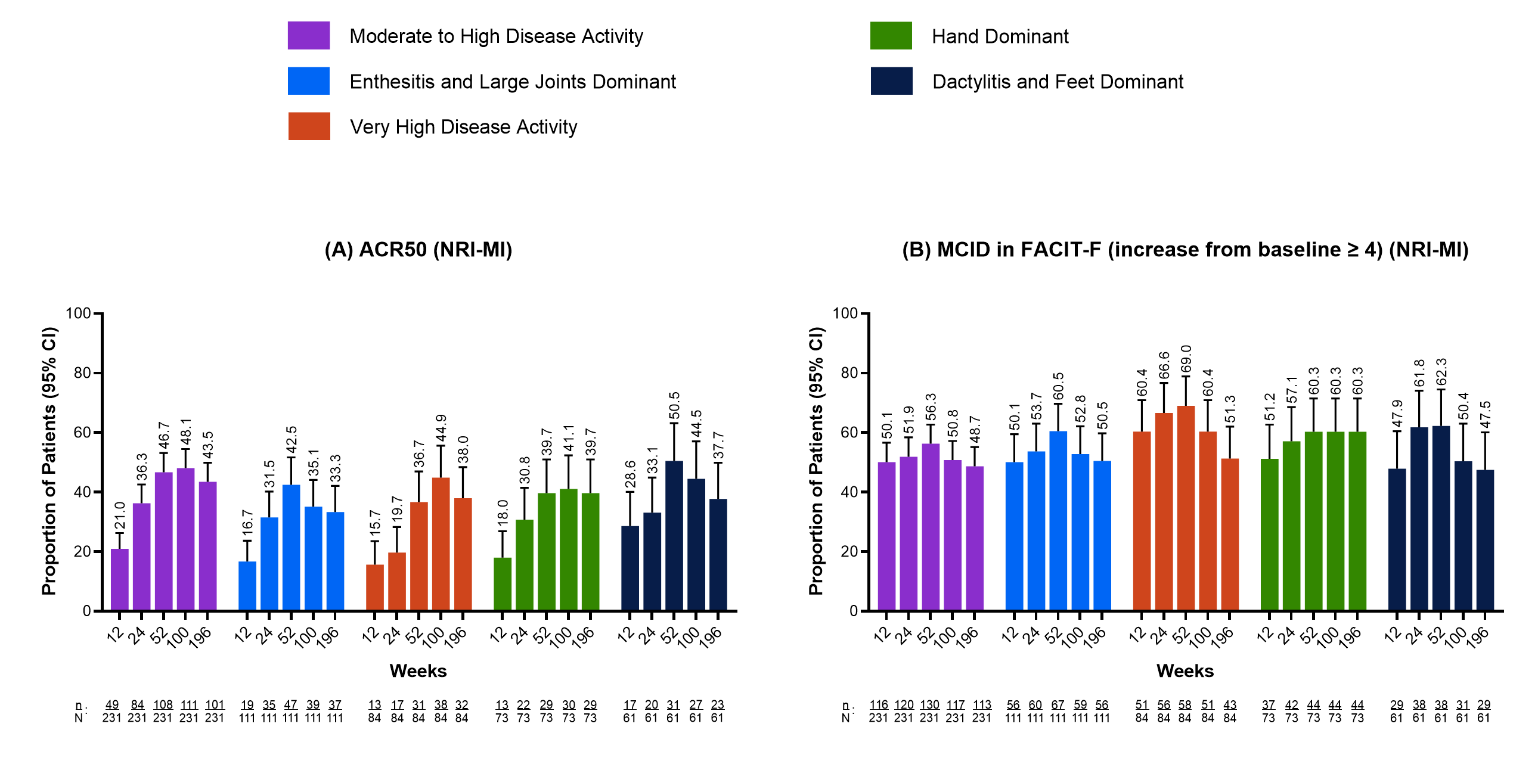
**

**Supplemental Table 1. Variables With Excess Missing Data and High Collinearity Excluded From the** **Unsupervised Machine Learning Model**

| **Variable Name** | **Variable Description** | **Reason for Exclusion** |
| --- | --- | --- |
| STRDAM | Structural damage defined as ≥ 1 erosion on radiograph as determined by central imaging review | Missing data of 23.5% (compared with ≤ 1.6% for all other variables) |
| NUMBTEND | Number of involved tender joints, not imputed (joints with missing data were not counted) | Highly correlated with variable TJC_68 (number of involved tender joints, used extrapolation to impute joints that could not be assessed); Pearson correlation coefficient = 0.9997 |
| NUMBSWOL | Number of involved swollen joints, not imputed (joints with missing data were not counted) | Highly correlated with variable SJC_66 (number of involved swollen joints, used extrapolation to impute joints that could not be assessed); Pearson correlation coefficient = 0.9998 |
| DACTYL | Presence of dactylitis (binary data) | Overlap with NUMBDACT (number of joints affected by dactylitis; continuous data); continuous data provided more information than the binary representation of the data |

**Supplemental Table 2. Variables and Associated Categories for the Unsupervised Machine Learning Model**

| **Variable Categories** | **Variables** |
| --- | --- |
| Demographics | Age group, sex, BMI, smoking status, race, ethnicity, region, work status |
| Tender joint-related | Tender joint involvement (by joint, yes/no), TJC1, TJC2,…TJC68, number of tender joints involved |
| Swollen joint-related | Swollen joint involvement (by joint, yes/no), SJC1, SJC2,… SJC66, number of swollen joints involved |
| Psoriasis-related | Skin involvement, psoriasis location (head), psoriasis location (lower extremities), psoriasis location (trunk), psoriasis location (upper extremities), psoriatic spondylitis |
| Dactylitis-related | Dactylitis location (hand), dactylitis location (feet), number of joints affected by dactylitis |
| Enthesitis-related | Presence of enthesitis, enthesitis score, enthesitis location 1, enthesitis location 2,… enthesitis location 16 |
| Other | Pain VAS, FACIT-F, CRP, duration of PsA, structural damage |

BMI, body mass index; CRP, C-reactive protein; FACIT-F, Functional Assessment of Chronic Illness Therapy - Fatigue; PsA, psoriatic arthritis; SJC, swollen joint count; TJC, tender joint count; VAS, visual analog scale
